# Hippocampal adaptation to high altitude: a neuroanatomic profile of hippocampal subfields in Tibetans and acclimatized Han Chinese residents

**DOI:** 10.3389/fnana.2022.999033

**Published:** 2022-11-17

**Authors:** Lianqing Zhang, Jinli Meng, Hailong Li, Mengyue Tang, Zan Zhou, Xingning Zhou, Li Feng, Xiangwei Li, Yongyue Guo, Yuanyuan He, Wanlin He, Xiaoqi Huang

**Affiliations:** ^1^Huaxi MR Research Center (HMRRC), Functional and Molecular Imaging Key Laboratory of Sichuan Province, Department of Radiology, West China Hospital, Sichuan University, Chengdu, China; ^2^Psychoradiology Research Unit of the Chinese Academy of Medical Sciences (2018RU011), West China Hospital of Sichuan University, Chengdu, China; ^3^Department of Radiology, Hospital of Chengdu Office of People’s Government of Tibetan Autonomous Region (Hospital C.T.), Chengdu, China

**Keywords:** hippocampus, hypoxia, adaptation, high-altitude, Tibetan

## Abstract

The hippocampus is highly plastic and vulnerable to hypoxia. However, it is unknown whether and how it adapts to chronic hypobaric hypoxia in humans. With a unique sample of Tibetans and acclimatized Han Chinese individuals residing on the Tibetan plateau, we aimed to build a neuroanatomic profile of the altitude-adapted hippocampus by measuring the volumetric differences in the whole hippocampus and its subfields. High-resolution T1-weighted magnetic resonance imaging was performed in healthy Tibetans (TH, *n* = 72) and healthy Han Chinese individuals living at an altitude of more than 3,500 m (HH, *n* = 27). In addition, healthy Han Chinese individuals living on a plain (HP, *n* = 72) were recruited as a sea-level reference group. Whereas the total hippocampal volume did not show a significant difference across groups when corrected for age, sex, and total intracranial volume, subfield-level differences within the hippocampus were found. *Post hoc* analyses revealed that Tibetans had larger core hippocampal subfields (bilateral CA3, right CA4, right dentate gyrus); a larger right hippocampus–amygdala transition area; and smaller bilateral presubiculum, right subiculum, and bilateral fimbria, than Han Chinese subjects (HH and/or HP). The hippocampus and all its subfields were found to be slightly and non-significantly smaller in HH subjects than in HP subjects. As a primary explorational study, our data suggested that while the overall hippocampal volume did not change, the core hippocampus of Tibetans may have an effect of adaptation to chronic hypobaric hypoxia. However, this adaptation may have required generations rather than mere decades to accumulate in the population.

## Introduction

Hypobaric hypoxia, the dramatically decreased availability of oxygen at high altitudes, poses significant challenges to humans residing there. The Tibetan Plateau is one of the highest plateaus in the world, and native Tibetans are adapted to life and reproduction in such a hypoxic environment with their unique protective physiological traits and were studied as an evolution paradigm for the high-altitude adaptation (Petousi and Robbins, 2014). However, newcomers to Tibet from lowland areas suffer from the physical and cognitive influence of high-altitude exposure (Yan, 2014). Studies consistently report noticeable declines in cognitive function, especially impairments in short-term memory, in migrants to a plateau above an altitude of 6,000 m (Virués-Ortega et al., 2004; Chen et al., 2017). Recently, brain structural and functional alterations related to cognitive declines following chronic exposure (2 years) to high altitude in college freshmen were also reported, including decreased gray matter volume in subcortical areas, disrupted white matter integrity, and altered resting-state networks (Chen et al., 2017, 2019, 2021). This evidence raises further questions regarding whether and how the human brain can adapt to high-altitude environments and how long this process requires.

The hippocampus is of particular interest in neuroscience research for its important function in memory and because it is a highly plastic structure that is vulnerable to multiple factors, including hypoxia. Furthermore, the hippocampus consists of several histologically and functionally different subfields, and our previous work found distinctive hippocampal neuroanatomic profiles in psychiatric disorders (Hu et al., 2019; Zhang et al., 2019, 2020). These subfields could be heterogeneously affected by hypoxia. In rats, chronic hypobaric hypoxia induced apoptosis, neuronal pyknosis, cell shrinkage, and consequent intercellular vacuolization in the CA1 and CA3 subfields of the hippocampus (Maiti et al., 2007; Hota et al., 2008a), and therapy such as ceftriaxone, oxygen enrichment or hypothermia could rescue hippocampal neurons from excitotoxicity and memory impairments in chronic hypobaric hypoxia (Hota et al., 2008b; Cai et al., 2021; Ranjan et al., 2022). In human subjects, how the hippocampus reacts to chronic hypobaric hypoxia and whether it can adapt remain largely unknown due to difficulties in finding research samples and the lack of research tools. In patients with obstructive sleep apnea, which causes intermittent hypoxia during sleep, damage to the hippocampus was found with cortical thinning in the molecular layer of the dentate gyrus (DG), CA1, and some layers of the entorhinal cortex (Owen et al., 2019). However, patients with obstructive sleep apnea (OSA) suffer from intermittent hypoxia, which is different from the constant hypoxia that occurs at high altitudes.

There is also evidence showing that permanent residents at high altitudes have adapted to hypoxia *via* multiple biological processes, including altered hemoglobin levels, regional blood flow, and O_2_ utilization components of the O_2_ transport system (Moore, 2017). Among them, the rs1769793 variant reduces EGLN1 expression, a gene that regulates cellular hypoxic responses, in skeletal muscle and the hippocampus (Liu et al., 2020). In new migrants to the plateau, while regional homogeneity (ReHo) as measured by resting-state fMRI was decreased over the brain, increased ReHo was found in the hippocampus, suggesting a special response in the hippocampus compared to other brain regions (Chen et al., 2017). Again, none of this evidence provides information on whether the hippocampus adapts to high altitude.

Herein, to better understand the reaction and adaptation of the human hippocampus to high altitude, we aim to build a neuroanatomic profile of the hippocampus in a unique sample of healthy Tibetan individuals living at high altitude (HT, adapted population), Han Chinese individuals living at high altitude (HH, acclimatized newcomers), and Han Chinese individuals living on the plain (PH, as a sea-level reference). As in the aforementioned reports showing the toxic effects of hypoxia in pyramidal cells in the hippocampus, we hypothesized that: (1) the hippocampal volumes would be larger in the TH as a result of adaptation and would be smaller in the HH as a result of the toxic effect of hypoxia; and (2) these adaptive/toxic effects of hypoxia would be observed primarily in the core hippocampal subfields (CA and DG), which showed sensitivity to hypoxia.

## Materials and Methods

### Participants

The study was approved by the local Research Ethics Committee, and written informed consent was obtained prior to study participation. Residents at an altitude of more than 3,500 m were recruited at the Hospital of Chengdu Office of People’s Government of Tibetan Autonomous Region (Hospital C.T.) with a poster advertisement. The inclusion criteria were as follows: (1) long-term residence at a high altitude of more than 3,500 m above sea level and residence at such an altitude within the last year; (2) arrival in Chengdu (sea level) for brief travel; (3) age between 18 and 75 years; and (4) right-handedness. The exclusion criteria were as follows: (1) an acute state of stress; (2) head injury, stroke or any other significant medical or neurologic conditions; (3) pregnancy; (4) systemic medical illness thought to interfere with brain function; and (5) contraindications to magnetic resonance imaging. Ultimately, a total of 96 high-altitude residents were recruited and assigned to the Tibetan high-altitude group (TH, *n* = 72) and the Han Chinese high-altitude group (HH, *n* = 27) based on their ethnicity. In addition, healthy age- and sex-matched Han Chinese individuals living on the plain (HP, *n* = 72) was recruited as a sea-level reference group. These HP subjects were screened to confirm the absence of any history of high-altitude exposure, any significant physical conditions or psychiatric disorders. Their demographic data are reported in Table 1; no significant difference was found in age, sex ratio, education level, body mass index (BMI), red blood cell count (RBC), mean corpuscular hemoglobin (MCH) or mean corpuscular hemoglobin concentration (MCHC).

**Table 1:** Demographic and clinical data in Tibetans (TH) and in Han Chinese individuals living at High Altitude (HH) or on the Plain (HP).

	**TH (*n* = 72) Mean (S.D.)**	**HH (*n* = 27) Mean (S.D.)**	**HP (*n* = 72) Mean (S.D.)**	**F/χ^2^**	***p* value**
Age	43.65 (11.4)	46.04 (13.7)	41.86 (9.3)	1.494	0.228
Gender (male/female)	30/42	16/11	28/44	3.353	0.187
Educational level	11.52 (7.9)	12.88 (6)	16.21 (1.9)	2.385	0.105
BMI	24.48 (4.9)	22.64 (5.5)	22.7 (2.7)	2.941	0.056
RBC	4.63 (0.6)	4.68 (0.6)	4.86 (0.5)	1.613	0.205
MCH	30.32 (3.3)	30.01 (3.4)	30.12 (2.8)	0.079	0.924
MCHC	328.48 (12.2)	328.39 (15.7)	331.66 (12)	0.624	0.538
eTIV (mm)	1,506,685.95 (201,858.9)	1,493,662.61 (174,285.8)	1,450,198.27 (238,279.8)	1.308	0.273

Notes: BMI, body mass index; RBC, red blood cell count; MCH, mean corpuscular hemoglobin; MCHC, mean corpuscular hemoglobin concentration; eTIV, estimated total intracranial volume.

### Structural MRI data acquisition

MRI data were acquired using a Philips Achieva 3.0 T MRI system and an eight-channel phase array head coil. A high-resolution T1-weighted 3D spoiled gradient recall (SPGR) sequence was used [repetition time (TR) = 8.1 ms, echo time (TE) = 3.7 ms, flip angle = 12°, slice thickness = 1.0 mm]. The field of view was 256 × 256 mm^2^ with an acquisition matrix = 256 × 256, which yielded an actual voxel size = 1 × 1 × 1 mm^3^. Foam padding and earplugs were used to reduce head motion and scanner noise.

### Volumetric analysis

The hippocampal subfield volume analysis protocol followed the protocol used in our previous work (Hu et al., 2019; Zhang et al., 2019, 2020). Anatomic images were automatically segmented using FreeSurfer software (V. 7.1.1)^1^. The recon-all FreeSurfer analysis pipeline was applied. Briefly, T1-weighted images were corrected for head motion and transformed into Talairach space, and normalization and skull-strip procedures were performed (Sled et al., 1998; Fischl et al., 2002, 2004; Reuter et al., 2010). The estimated total intracranial volume (eTIV) of each subject was collected.

**Figure 1:**
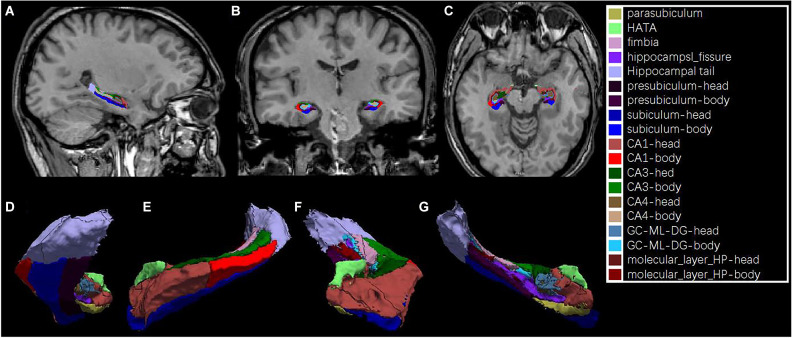
An example of hippocampal subfield segmentation in healthy Han Chinese individuals living on the plain. Upper row: **(A)** sagittal, **(B)** coronal and, **(C)** axial view of the hippocampus in the brain; Lower row: 3D reconstruction of the hippocampal subfields in **(D)** dorsal, **(E)** lateral, **(F)** frontal and, **(G)** medial view.

Hippocampal subfield segmentation was performed using a module in FreeSurfer software that was designed for the purpose; the module employs a tetrahedral mesh-based probabilistic atlas built from manually delineated hippocampus in both *in vivo* and *ex vivo* data (Iglesias et al., 2015). Using this algorithm, the overall volumes of the bilateral hippocampus and their subfields were obtained. Two sets of segmentations with different levels of hierarchy were generated: (1) head, body, and tail (usually referred to as subregions); and (2) CA1, CA3 (which contains CA2), CA4, the molecular and granule cell layers of the dentate gyrus (GC-ML-DG), the molecular layer, subiculum, presubiculum, parasubiculum, fimbria, and hippocampus–amygdala transition area (HATA). An example of the segmentation for a healthy subject is shown in Figure 1. All segmentation was visually verified following a quality control protocol that is similar to the ENIGMA protocol^2^. In brief, the segmentation of each subject was independently visually checked by two coauthors (LZ and MT), and any subject with segmentation results judged to be incorrect was excluded; no such segmentation failures occurred.

### Statistical analysis

A multivariate analysis of covariance (MANCOVA) test was used to test for volume differences in overall hippocampal volume and its subregions/subfields across TH, HH and HP groups, with age, sex and eTIV as covariates, and the false discovery rate (FDR) method was used to correct multiple hypothesis testing issues across subregions/subfields. For subfields that showed significance after FDR correction, *post-hoc* tests were employed to determine where the difference between groups was. The partial eta squared (η^2^) was calculated to estimate effect sizes. Partial correlation analyses with age, sex, and eTIV as covariates were conducted between volumes and BMI, RBC, MCH, and MCHC levels to find potential associations.

## Results

**Figure 2:**
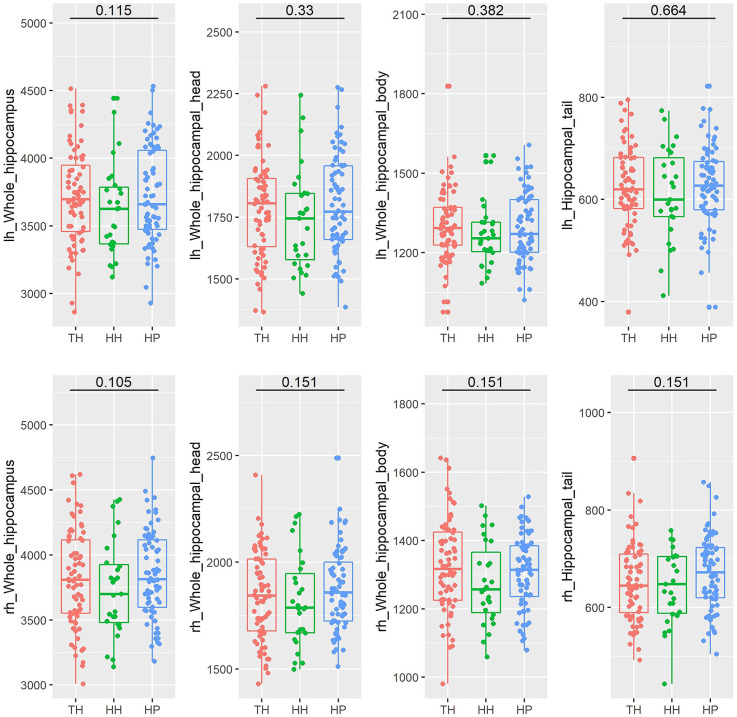
Box plots of the volumes of the entire hippocampus and its subregions (head, body, and tail) in healthy Tibetan individuals living at high altitude (TH, adapted population, shown in red), Han Chinese individuals living at high altitude (HH, acclimatized newcomers, shown in green), and Han Chinese individuals living on the plain (HP, sea-level reference, shown in blue). The annotations show the FDR-corrected *p* values. lh, left hemisphere; rh, right hemisphere.

**Figure 3:**
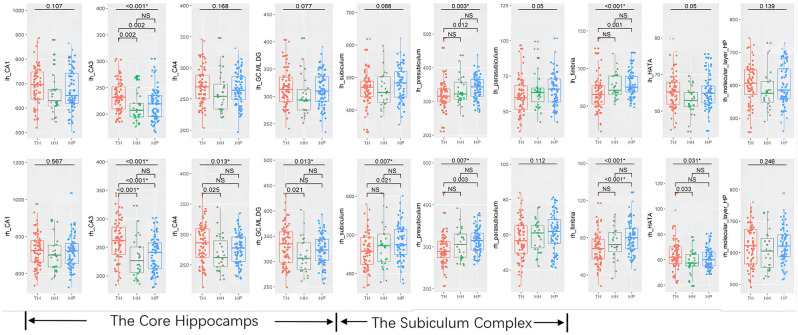
Box-plots of subfields of the hippocampus in healthy Tibetan individuals living at high altitude (TH, adapted population, shown in red), Han Chinese individuals living at high altitude (HH, acclimatized newcomers, shown in green), and Han Chinese individuals living on the plain (HP, sea-level reference, shown in blue). The annotations show the FDR-corrected *p* values, and * indicates statistical significance. lh, left hemisphere; rh, right hemisphere. NS, not significant.

The whole hippocampal volume or any of the subregions, including the head, body or tail, did not show a significant difference across groups when corrected for age, sex, and ICV (Table 2, Figure 2). However, subfield-level differences within the hippocampus were found. The “core hippocampus,” including the CA subfields and the GC-ML-DG, was largest in the TH group and smallest in the HH group, while the subiculum complex, fimbria, and HATA were smallest in the TH group, except for the right subiculum and right HATA (Table 2, Figure 3). These volume alterations were statistically significant in the subfields of the bilateral CA3, right CA4, right GC-ML-DG, right subiculum, bilateral presubiculum, bilateral fimbria, and right HATA. No associations were found between volumes and BMI, RBC, MCH or MCHC levels.

**Table 2:** Multivariate Analysis of Covariance (MANCOVA) and *post hoc* analysis of hippocampal volume in Tibetans (TH) and in Han Chinese individuals living at High Altitude (HH) or on the Plain (HP).

	**HP (*n* = 72) Mean (S.D.)**	**HH (*n* = 27) Mean (S.D.)**	**TH (*n* = 69) Mean (S.D.)**	**F**	**Effect size**	**p_FDR_**	**HH vs. HP**	**HH vs. TH**	**TH vs. HP**
Hippocampus							
Left									
Overall volume	3,725.65 (353.7)	3,618.77 (341.6)	3,722.35 (358.6)	1.519	0.014	0.222	-	-	-
Head	1,803.21 (197.5)	1,743.25 (207.8)	1,788.43 (200.3)	1.448	0.015	0.434	-	-	-
Body	1,294.39 (130.9)	1,264.22 (115.2)	1,309.24 (135.1)	1.25	0.012	0.434	-	-	-
Tail	628.05 (77.2)	611.31 (90.1)	624.68 (81.5)	0.27	0.003	0.764	-	-	-
CA1	673.07 (84.9)	656.22 (83.9)	689.97 (81.9)	1.89	0.02	0.192	-	-	-
CA3	218.41 (30)	212.79 (27)	235.38 (31)	8.663	0.084	<0.001*	0.407	0.002*	0.002*
CA4	266.45 (26.8)	260.32 (31.3)	271.18 (29)	1.565	0.016	0.212	-	-	-
Molecular layer	600.67 (61.8)	582.99 (58.7)	605.31 (61.5)	1.582	0.016	0.212	-	-	-
GC-ML-DG	312.92 (31.5)	301.49 (36.9)	316.96 (33.9)	2.56	0.026	0.120	-	-	-
Subiculum	481.1 (53.4)	464.8 (55.3)	468.73 (50.7)	2.51	0.026	0.120	-	-	-
Presubiculum	340.12 (35.3)	331.82 (41.4)	321.4 (43.3)	5.9	0.062	0.010*	0.356	0.356	0.017*
Parasubiculum	65.43 (11.3)	64.89 (13.4)	60.91 (13)	3.253	0.036	0.082	-	-	-
Fimbria	79.07 (16.9)	76.1 (17)	66.85 (23.2)	9.614	0.094	<0.001*	0.506	0.061	0.001*
HATA	60.38 (8.5)	56.04 (7.2)	60.99 (9.4)	3.793	0.04	0.062	-	-	-
Right									
Overall volume	3,850.33 (331.9)	3,739.83 (366.1)	3,821.97 (380.5)	1.547	0.014	0.216	-	-	-
Head	1,872.66 (188.2)	1,820.82 (213.7)	1,841.25 (208.5)	1.443	0.015	0.239	-	-	-
Body	1,306.83 (107.7)	1,275.99 (120.1)	1,326.58 (138.8)	1.829	0.017	0.239	-	-	-
Tail	670.84 (76.6)	643.02 (75.7)	654.14 (81.1)	1.512	0.015	0.239	-	-	-
CA1	718.83 (86.1)	707.27 (98.1)	722.83 (89.4)	0.317	0.003	0.729	-	-	-
CA3	237.96 (30.5)	232.14 (34.6)	262.3 (35.2)	13.971	0.128	<0.001*	0.438	<0.001*	<0.001*
CA4	276.59 (24.6)	268.03 (29.3)	285.48 (32)	4.822	0.046	0.015*	0.187	0.024*	0.100
Molecular layer	624.46 (56.9)	607.93 (64.8)	621.14 (66.2)	1.038	0.01	0.396	-	-	-
GC-ML-DG	323.36 (29.8)	311.24 (35)	332.76 (38.5)	4.924	0.048	0.015*	0.121	0.020*	0.121
Subiculum	480.89 (45.2)	472.48 (57.2)	459.44 (47.5)	4.995	0.05	0.015*	0.440	0.353	0.027*
Presubiculum	314.69 (29.2)	305.36 (36)	294.61 (38.2)	6.613	0.071	0.007*	0.229	0.229	0.002*
Parasubiculum	61.2 (10.7)	59.74 (8.8)	57.21 (11.4)	2.598	0.03	0.098	-	-	-
Fimbria	80.38 (16.8)	74.63 (17.2)	68.19 (17.2)	10.899	0.107	<0.001*	0.136	0.136	<0.001*
HATA	61.13 (8)	58 (9)	63.86 (12.2)	3.64	0.041	0.040*	0.172	0.034*	0.165

Note: TH, Tibetan individuals living at high altitude; HH, Han Chinese individuals living at high altitude; HP, Han Chinese individuals living on the plain. GC-ML-DG, the molecular and granule cell layers of the dentate gyrus; HATA, hippocampus–amygdala transition area. *Indicates that the significance survived false discovery rate correction for multiple comparisons.

*Post hoc* analyses revealed that Tibetans had a larger core hippocampus and right HATA than Han Chinese individuals. Specifically, the TH group showed larger CA3 bilaterally than the HP (*post hoc*: left *p* = 0.002, right *p* < 0.001) or HH (*post hoc*: left *p* = 0.002, right *p* < 0.001) groups. The right CA4 (*post hoc* *p* = 0.024) and right GC-ML-DG (*post hoc* *p* = 0.02) were found to be significantly larger in the TH group than in the HH group (Table 2, Figure 3). For the subiculum complex, the bilateral presubiculum (*post hoc*: left *p* = 0.017, right *p* = 0.002) and right subiculum (*post hoc*: right *p* = 0.027) were found to be significantly smaller in the TH group than in the HP group (Table 2, Figure 3). Finally, bilateral fimbria were significantly smaller in the TH group than in the HP group (*post hoc*: left *p* = 0.001, right *p* < 0.001). No differences were found to be significant between the HH and HP groups.

## Discussion

To our knowledge, this is the first study to document hippocampal volume in normal residents at high altitudes. Notably, we included Tibetan and Han Chinese individuals living at high altitudes to find possible hippocampal adaptation to chronic hypoxia at different time scales (generational or lifetime) and recruited a group of normal Han Chinese subjects as the sea-level reference. Moreover, with subfield-level analyses, we are able to detect subfield-level hippocampal differences that would not be revealed by the global volume. We found that: (1) the Tibetans (generationally adapted to chronic hypoxia) had larger subfields in the “core hippocampus” (CA3, CA4, and GC-ML-DG) and smaller subfields of subiculum, presubiculum, and fimbria than Han subjects, suggesting an effect of adaptation that could be protective to the “core hippocampus”; and (2) Meanwhile, Han Chinese individuals who are residents on the plateau for decades were observed to have slightly but not significantly smaller hippocampal volume across subfields compared with HP, showing a mild effect of chronic hypobaric hypoxia on hippocampal volume that is not subregional specific.

Tibetans appear to have lived at high altitude the longest over the world among three human populations that have lived at high altitudes for millennia, suggesting that Tibetans have had more time and opportunity for natural selection in response to a hypoxic environment than any other high-altitude human population (Petousi and Robbins, 2014). Our reported neuroanatomic profile of the hippocampal subfield volume could indicate an effect of human hippocampal adaptation to the high-altitude environment. The larger “core hippocampus” found in Tibetans could be a result of their distinctive physiological traits or resistance to certain pathophysiological processes in high-altitude environments. These physiological traits mainly involve cardiovascular, respiratory, and hematopoietic physiology, which affect convective oxygen transport (Petousi and Robbins, 2014).

Multiple genomic loci that underwent natural selection in Tibetans were found, e.g., EGLN1 and EPAS1 encode major components of the hypoxia-inducible factor transcriptional system, which has a central role in oxygen sensing and coordinating an organism’s response to hypoxia (Lorenzo et al., 2014; Petousi and Robbins, 2014). A previous study reported that EGLN1 variants found to be associated with higher VO_2_max (maximal oxygen consumption, a metric commonly used to evaluate exercise performance) in hypoxia in Peruvian Quechua individuals could significantly reduce EGLN1 expression in the skeletal muscle and hippocampus compared with the other 48 tissues, including other brain tissues from other brain regions (Liu et al., 2020).

This finding could indicate that the hippocampus is more sensitive to increased oxygen transport efficiency than other brain regions. For example, aerobic exercise training could increase the hippocampus volume or effectively reverse age-related loss in hippocampal volume in human subjects, and this effect in the hippocampal volume is significantly correlated with the improvement in VO_2_max (Erickson et al., 2011; Aghjayan et al., 2021). Strikingly, with higher exercise performance (measured with exercise workload), Tibetans exhibit lower hemoglobin concentration and VO_2_max than acclimatized newcomers (Ge et al., 1994; Garruto et al., 2003; Petousi and Robbins, 2014). These findings indicate that Tibetans may have adapted in their own way to use oxygen more efficiently. Either way, elevated oxygen transport or consumption could be beneficial to the hippocampus, leading to increased hippocampal volume in its core subfields.

The volume increase in Tibetans was found selectively in the “core hippocampus,” especially the CA3, CA4, and DG. These subfields mainly consist of pyramidal neurons, and neurogenesis was found in these subfields. Meanwhile, a volume decrease was found in subfields of the (pre)subiculum and fimbria, which are widely considered the input/output structure of the hippocampus (Roddy et al., 2019). Such selectivity suggests that there are regionally dependent molecular pathways in Tibetans. Animal studies demonstrated that in adult rodents, aerobic exercise could increase synaptic strength and plasticity in the hippocampus and promote neurogenesis with the DG (van Praag et al., 1999; Rhodes et al., 2003; van Praag, 2008; Hötting and Röder, 2013). This suggests that a higher VO_2_max may promote neurogenesis in the hippocampus.

Meanwhile, hippocampal neurons are also sensitive to the toxic effect of hypoxia. It was reported that hypobaric hypoxia induced apoptosis in the CA1 region and damaged hippocampal pyramidal neurons in rats (Maiti et al., 2007; Hota et al., 2008a). We hypothesize that the protective physiological mechanism to hypoxia adapted in Tibetans is more sensitive in neuron types in the subfields of the “core hippocampus” but less sensitive in other subfields, which resulted in a unique neuroanatomic profile of the hippocampus in Tibetans found in the current study.

Although the difference was not statistically significant, the hippocampal volume was slightly smaller across all its subfields in acclimatized Han Chinese lowlanders who migrated to a high altitude compared with their sea-level counterparts. This is partly in line with our hypothesis that hypoxia is toxic to the hippocampus and may lead to volume decreases in highland newcomers. The observed hippocampal neuroanatomic profile indicates an effect of long-term acclimatization in lowlanders who migrate to high altitudes. A previous study showed significantly lowered accuracy in memory tests and longer reaction times after exposure, together with markedly decreased volumes or gray matter volumes in other subcortical nuclei but not the hippocampus in lowlanders who relocated to the Tibetan Plateau for 2 years (Chen et al., 2017, 2019). These results suggest that even if hypoxia is toxic to the hippocampus (as discussed above), the effect may not be substantial enough for its volume decrease to reach statistical significance.

As we recruited a sample of lowlanders who migrated to high altitude for longer than 2 years, the effect of their acclimatization to hypoxia could further normalize hippocampal alterations under hypoxia. For example, in rats, accumulation was found to promote neuronal regeneration together with memory/cognitive function recovery in the hippocampus during exposure to a chronic high-altitude hypoxic environment (Chen et al., 2022). Another possible explanation is that the effect of hypoxia on hippocampal neurons could be complex. While the hippocampus is more susceptible to acute hypoxic injury (Zhang et al., 2022), chronic intermittent hypobaric hypoxia could restore hippocampal function and may serve as a potential treatment for epilepsy-induced cognitive impairments (Sun et al., 2020). Taken together, the outcome of brain hypoxia may depend on the type, severity, exposure duration, and frequency of hypoxia (Burtscher et al., 2021). However, hippocampal neuron changes across time under chronic continuous hypobaric hypoxia on the plateau remain to be clarified.

Our study has some limitations. First, while it is interesting that we documented a unique neuroanatomic profile of the hippocampus in Tibetans, our result may not be generalizable to other highland populations (Andeans on the Andean Altiplano and Ethiopians on the Simien Plateau) because Tibetan populations may have followed different evolutionary pathways with their unique culture and daily habits (Beall, 2000). Second, the HH group, the Han Chinese individuals who migrated to a high altitude, had a relatively small sample size compared with the TH and HP groups and thus reduce the statistical power. Potential confounds related to migration (e.g., smoking history, stress from migrating, etc.) could contribute to hippocampal volume differences, hence, the results should be interpreted with caution. Finally, we did not report cognitive assessments for Tibetans which could be informative. We had tried to collect such data by translating these tests into local dialog. However, we found that more than 50% of Tibetan subjects could not finish the test and we believe that finding validated cognitive tests for Tibetan is a topic worth further study.

## Data Availability Statement

The data presented in the current study are available from the corresponding authors upon reasonable request.

## Ethics Statement

The studies involving human participants were reviewed and approved by Ethics committee of Hospital of Chengdu Office of People’s Government of Tibetan Autonomous Region (Hospital C.T.). The patients/participants provided their written informed consent to participate in this study.

## Author Contributions

HL, YG, WH, and XH conceived and designed the study. ZZ, XZ, and LF collected MRI data and performed the quality control. LZ, HL, and MT analyzed the data and performed statistical analyses. LZ, JM, and XL interpreted the statistical outcomes and wrote the manuscript. All authors contributed to the article and approved the submitted version.

## Funding

This study was supported by the Science and Technology Project of Sichuan Province (Grant No. 2021YJ0161), the Medical Research Project of Sichuan Province (Fund No. Q20042), the Science and Technology Project of Tibet Autonomous Region: (“The central government guides local projects,” Grant No. XZ202102YD0032C), the 1.3.5 Project for Disciplines of Excellence, West China Hospital, Sichuan University (ZYJC21041), The Research Project of Shanghai Science and Technology Commission (20dz2260300), and The Fundamental Research Funds for the Central Universities.

## Footnotes

^1^ http://surfer.nmr.mgh.harvard.edu/

^2^ http://enigma.ini.usc.edu/
